# Psychological resilience and positive coping styles among Chinese undergraduate students: a cross-sectional study

**DOI:** 10.1186/s40359-020-00444-y

**Published:** 2020-08-06

**Authors:** Yu Wu, Wenzhou Yu, Xiuyun Wu, Huihui Wan, Ying Wang, Guohua Lu

**Affiliations:** 1 Weifang Medical University, 7166 Baotong West Street, Weifang, 261053 Shandong Province China; 2 Heart Center, Sunshine Union Hospital, 9000 Yingqian Street, Weifang, 261061 Shandong Province China

**Keywords:** Resilience, Coping styles, Undergraduates, Psychological education

## Abstract

**Background:**

Psychological resilience and coping strategies have been found to be related to various psychological and mental health problems. Evaluations of the relationship between resilience and coping style among university students are important for developing effective health promotion strategies focused on resilience intervention to benefit students’ health and well-being. The relationship between psychological resilience and coping styles has usually been examined among adults and patients. Very few studies have investigated the relationship between resilience and coping style in university students. The present study aimed to investigate the associations between psychological resilience, students’ characteristics (gender, major and grade) and coping styles among undergraduate students.

**Methods:**

A cross-sectional survey was conducted among undergraduate students in Shandong Province, China. Undergraduate students were randomly selected from 6 universities in 3 cities of the province using a stratified random sampling method. The questionnaire included questions on the participants’ demographic information, including gender, grade and major, measures of psychological resilience and coping style. Coping style was measured by the Simplified Coping Style Questionnaire (SCSQ). The Asian Resilience Scale (ARS) was applied to evaluate undergraduates’ psychological resilience. Multivariable regression analysis was used to examine the relationships between resilience, students’ characteristics and positive coping styles.

**Results:**

A sample of 1743 undergraduates was analysed. The mean psychological resilience score was 70.41. The mean score for positive coping style was 24.72. Multiple regression analysis showed that three factors of psychological resilience, mood control, self-plasticity and coping flexibility, were all significant factors for positive coping styles (regression coefficient = 0.34, 0.35, 0.14, *p* < 0.01 for the three factors, respectively). Medical students and females had higher scores for positive coping styles than non-medical students and males (*p* < 0.01).

**Conclusions:**

The research revealed that females and medical students are more likely than males and non-medical students to adopt positive coping styles. Higher psychological resilience is associated with a better positive coping style. The findings suggest that psychological education and health promotion programmes that target strengthening psychological resilience among undergraduate students may help foster positive coping styles to benefit their mental health and psychological well-being.

## Background

University life is a process of adaption to changes at the juncture between school and social life, and undergraduate students in particular are in a critical transitional period [1]. The changes in this period affect the psychosocial and emotional development and mental health of undergraduates [2]. Previous studies have shown that in the early years of university study (freshman and sophomore), undergraduate students experience stress that mostly involves the re-establishment of interpersonal relationships and dealing with heavy academic pressure [3]. In the latter period of university study (years 3 to 5), the sources of stress for undergraduates are more likely to be anxiety and frustration about seeking a job or failure to find one [1]. Some undergraduates are prone to various psychological problems during this period because of increasingly heavy academic pressure and the difficulty of adapting to changes in university life and social communications. The psychological problems of undergraduates are acknowledged as a critical public health concern [4–7]. The mental health state of undergraduates is frequently labelled a ‘crisis’ by researchers as the demand among undergraduates for mental health disorder counselling and treatment services has rapidly increased [8]. The evidence from systematic reviews and primary studies indicates that over the past several decades, the prevalence of depression and other mental health disorders (e.g., anxiety, stress) among college and university students has been increasing worldwide [9–11].

The psychological problems of Chinese undergraduates are also serious [12, 13]. A survey of 126,000 Chinese undergraduates showed that approximately 16–30% of these students suffered from various mental health problems [13]. In a cohort study of university graduate students from Beijing during 2007–2009, the number of graduate students with mental health problems increased yearly, at 21.4, 23.6 and 29.23%, respectively [14]. Depression, anxiety and other negative emotions not only have a very significant impact on the psychological status of undergraduates but also lead to more serious consequences, such as suicide and violent behaviours [1, 15].

Psychological resilience has been defined by the American Psychological Association as ‘a process of good adaptation in the face of adversity, trauma, tragedy, threats or other significant sources of stressors such as family and relationship problems, serious health problems or financial problems’ [16]. It can be viewed as a measure of stress coping ability in response to adversity and is used as a target in the treatment of depression, anxiety and stress problems [17]. Previous research has shown that low psychological resilience is related to a number of mental health problems among patients, such as anxiety, depression and stress [18–20]. Among university and college students, studies have documented that improving psychological resilience can buffer the negative emotions of stress and contribute to students’ success in academic performance [21], improve students’ sense of well-being [22], and enable them to better cope with stressful events [23]. The promotion of resilience has been demonstrated to have positive effects on reducing depression among college students [24] and on reducing mental health problems (e.g., depressive and anxiety symptoms, stress) among children and adolescents [25, 26].

Coping styles refer to the cognitive and behavioural changes that result from the management of an individual’s specific external/internal stressors [27, 28]. Researchers have proposed three distinct types of coping styles: problem-focused coping, emotion-focused coping and avoidance coping [27, 29, 30]. Problem-focused coping is a task-oriented coping style that attempts to alter stressful situations with active efforts to solve the problem or reduce its negative impact. Emotion-focused coping aims to diminish stress events through emotional responses such as self-blaming, anger or self-preoccupation. Avoidance coping involves attempts to avoid stressful situations via social distraction or escape from the situation rather than actively facing and dealing with it. Psychological problems are affected by coping methods. A number of studies have demonstrated that coping style is associated with psychological health and well-being. Positive coping styles are related to higher levels of positive cognitive and behavioural adjustments in the face of stressful events and to a decreased risk of anxiety and depression [31, 32]. It has been reported that college students who were more optimistic and used positive coping methods were more willing to participate in social activities and to manage the negative impact of stress [33].

Although extensive research has examined the relationship between resilience and coping styles separately with regard to a number of psychological problems among university or college students [21, 22, 24, 31, 33, 34], few studies have investigated the relationship between resilience and coping styles among undergraduate students [29, 35]. Resilience and coping are related but different constructs with respect to their impact on behavioural changes. Coping refers to cognitive and behavioural strategies to handle and manage stressful events or negative psychological and physical outcomes [30], while resilience refers to the adaptive capacity to recover from stressful situations in the face of adversity [36]. The majority of previous studies have examined the relationship between resilience and coping among adults and patients [37, 38], and the relationship between resilience and positive coping among university and college students has been previously reported [29, 35]. However, the nature of the relationship between resilience and coping style has not yet been clearly established, with inconsistent findings reported in the literature. Evidence suggests that resilience is positively related to problem-focused coping and negatively related to emotion-focused coping [29, 39]. Some studies have found that coping style mediates the relationship between resilience and psychological well-being [29], while other studies have observed that coping style predicts resilience, and resilience may mediate the relationship between coping and psychological well-being or negative symptoms [37, 38]. However, a limitation of previous studies is that they did not examine the effect of resilience on coping with regard to a specific component of resilience (e.g., mood control, self-plasticity). This analysis would help to disentangle the relationship between each factor or subdimension of resilience and coping outcomes. More importantly, there is a paucity of relevant studies among university students in China. This study fills this gap by exploring the relationship between resilience and coping styles. Studies that have incorporated resilience intervention to enhance positive coping strategies among university students have shown that a higher level of resilience was related to more effective coping strategies, including better problem solving and less avoidant coping [36]. A better understanding of the relationship between resilience and coping styles among undergraduates is crucial for the development of effective health promotion strategies targeting resilience intervention and its positive impact on active coping to enhance the health and psychosocial well-being of university students.

Prior research has documented that the demographic characteristics of students, such as gender, major and grade level, are associated with coping styles [40–44]. There is some evidence that women are more likely to choose emotional-based or avoidance coping styles, whereas men use more problem-focused methods to handle stressful situations [41, 42]. However, the precise pattern of gender differences in coping styles among university or college students has yet to be studied. Some studies have shown no gender difference in the use of problem-solving or avoidance coping [40] or have found that women are more likely to use active coping and social support and to employ problem-focused coping [40, 45]. It is also important to investigate the influence of different majors and grade levels on coping styles. Some researchers have noted that older students are more likely to use positive coping strategies than younger students [43], and university students of different majors (e.g., medical and non-medical degree) tend to engage in different coping styles [44, 46]. Overall, there is a lack of studies that examine the relationship between coping style by major and years of study among university students, and there is no consensus in the findings due to sparse literature. To the best of our knowledge, little research has been conducted to compare coping styles between medical and non-medical undergraduate students as most existing studies have used samples of students in a single discipline (e.g., medical students only).

The purpose of this study was to examine the relationship between psychological resilience and coping styles and to investigate the influence of the demographic characteristics (gender, major and grade) of undergraduate students on psychological resilience and coping styles.

According to previous studies, we hypothesized that high levels of resilience would be associated with a better positive coping style, and female students would have higher positive coping style scores than male students. We expected that medical undergraduate students would score higher on positive coping styles than non-medical students.

## Methods

### Participants and data collection

This was a cross-sectional study. Undergraduate students were randomly selected from six universities in three cities of Shandong Province in China using a stratified random sampling method. The sampling scheme was stratified by the type of university and faculty, and classes were randomly selected within the strata. Students consented to participate in the survey and had to be able to understand and independently complete the questionnaire. Data collection was conducted at the universities between November 2014 and February 2015. The paper-based questionnaires were distributed to the students by research assistants, and the students completed the questionnaires in pen. Initially, 1746 undergraduate students consented to participate in the study and received the questionnaires. Of them, 1743 students completed the questionnaire, resulting in a response rate of 99.8%. The questionnaire included questions on students’ background information (gender, grade, major), the Asian Resilience Scale (ARS), and the Simplified Coping Style Questionnaire (SCSQ).

### Measures

#### The Asian resilience scale

The Chinese version of the Asian Resilience Scale questionnaire [47] was used in this study. The ARS was revised by Lu et al. [47] and consists of 19 items covering three subscales: self-plasticity, mood control and coping flexibility. Each item is rated on a five-point Likert scale ranging from 1 ‘strongly disagree’ to 5 ‘strongly agree’. The total score of the ARS ranges from 19 to 95, with higher scores indicating greater resilience. The scale has been previously tested in undergraduate and middle school students with good reliability and validity [47–49].

#### The simplified coping style questionnaire

The 20-item Simplified Coping Style Questionnaire, which is based on the Ways of Coping Questionnaire (WCQ) by Folkman and Lazarus [27], was developed and revised by Xie to be suitable for use in Chinese populations [50]. Students were asked how often they adopt the coping style. Each item is rated on a four-point Likert scale ranging from 0 ‘never’ to 3 ‘very often’. Two factors were extracted from the 20 questions of the questionnaire, which were defined as positive and negative coping styles. The Simplified Coping Style Questionnaire-Positive Styles (SCSQ-PS) has 12 items. Items of the SCSQ-PS include actively coping with setbacks by talking with others, seeking social support and advices from families, friends and relatives, learning from others’ experiences, changing negative thoughts and taking a positive view for stress, self-controlling hopelessness, sadness and anger, finding alternative solutions, and participating in physical and recreational activities. The total score of the SCSQ-PS ranges between 0 and 36, with higher scores indicating greater positive coping styles. The SCSQ has been widely used in Chinese population samples with good internal consistency reliability (Cronbach’s alpha of 0.89) [51].

#### Demographic characteristics of students

The students’ demographic characteristics included gender, age, major and study year (grade). The students’ major was categorized as medical and non-medical, and grade was grouped as junior (study years 1–2) and senior (study year 3–5).

### Statistical analyses

Descriptive analyses were conducted to present the frequency and percentage of the background characteristics of the undergraduates, including gender, grade level and major. Mean and standard deviation (SD) were used to present self-reported resilience and coping style scores by the background variables. The reliability of the Asian Resilience Scale and the SCSQ-PS was assessed by the internal consistency with Cronbach’s alpha coefficient. A confirmatory factor analysis (CFA) was used to examine the factor structure of the ARS that was previously established by Lu et al. [47]. The goodness of fit of the CFA model was tested using the following fit indices: RMSEA (the root-mean-square error of approximation), GFI (the goodness-of-fit index) and CFI (the comparative fit index). An RMSEA equal to or smaller than 0.08 and GFI and CFI values greater than 0.90 indicate an acceptable model fit [52]. To compare differences in resilience and coping style between groups by the students’ gender, grade and major (medical students versus non-medical students), independent-sample t-tests were used to test the statistical significance of the results.

Pearson’s correlation analysis was used to explore the bivariate association between the subscales of psychological resilience, coping styles, and social background characteristics. A multivariable linear regression with the stepwise method was used to verify the significant factors that predicted positive coping styles. The factors of resilience and demographics that showed a statistically significant correlation with coping style outcome (*p* ≤ 0.05) in the t-test and correlation analysis were selected for multiple linear regression analysis to delineate their associations with positive coping style. In the multiple linear regression model, coping style was entered as the dependent variable, and other factors were treated as predictor variables. Univariate regression analysis was used to test the interaction effects between the subscales of resilience and the gender, major and grade of the participants. In the analysis, the categorical variables were coded as follows: gender (male = 0, female = 1), major (medical = 0, non-medical = 1) and grade (junior = 0, senior = 1). All statistical tests were two-sided, and a *p* value< 0.05 was defined as the level of significance. SPSS software (Inc., Chicago, IL, USA) version 17.0 was used for the statistical analysis.

### Ethics approval

This study was approved by the ethics committee of Weifang Medical University. All participants signed an informed consent form prior to the administration of the questionnaires.

## Results

### Description of the social background characteristics

**Fig. 1:**
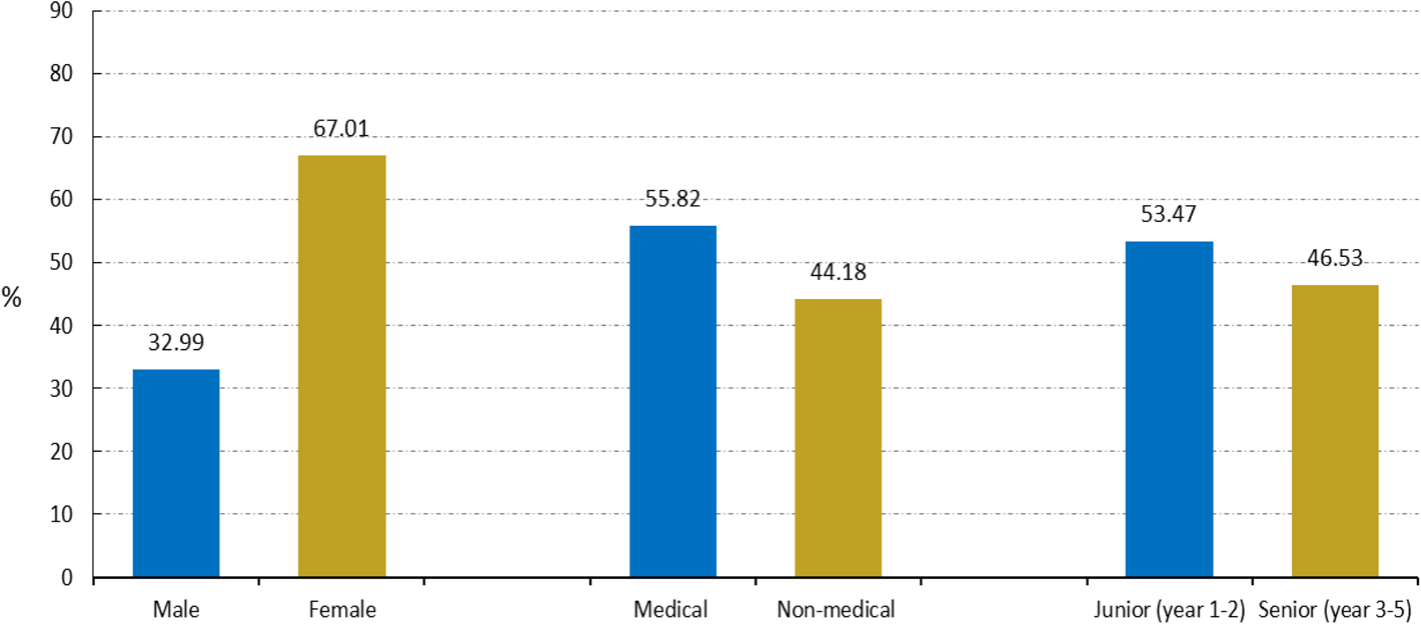
Frequency distribution (%) of the participants by gender, major and grade level (*N* = 1743)

The study sample included 1168 (67.01%) females and 575 (32.99%) males, of whom 973 (55.82%) were medical students and 770 (44.18%) were non-medical students. The mean (SD) age of undergraduate students was 21.11 (1.68) years, ranging from 17 to 26 years. The medical students were from faculties and schools such as clinical medicine, nursing, stomatology, etc. The non-medical students were from faculties and schools such as marine technology, automation engineering, landscape architecture, etc. Of these undergraduates, 932 (53.47%) were junior students (freshman and sophomore), and 811 (46.53%) were senior students (university years 3 to 5). The background characteristics of the participants are presented in Fig. 1.

### Psychological resilience and coping styles

**Fig. 2:**
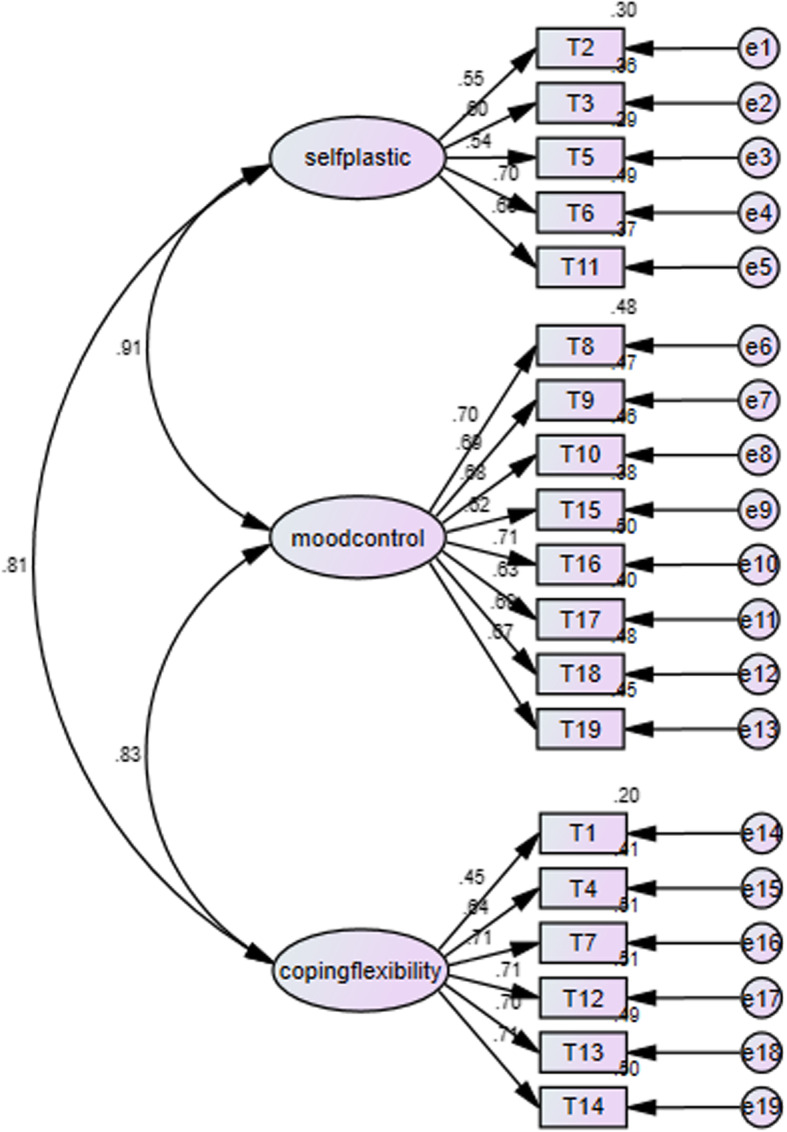
Confirmatory factor analysis for the 3-factor structure of the Asian Resilience Scale. Legend: T1–T19 are items in the Asian Resilience Scale; The numbers between the latent factors (self-plasticity, mood control and coping flexibility) and the items are correlation coefficients for the items; numbers between the three latent factors are correlation coefficients for the factors

The ARS and the SCSQ-PS showed high internal consistency reliability. The Cronbach’s alpha was 0.919 for the ARS and 0.8 for the SCSQ-PS scale. The Cronbach’s alpha for the three factors of the resilience scale was 0.738, 0.868 and 0.813 for self-plasticity, mood control and coping flexibility, respectively. The 3-factor CFA model for the ARS showed a good fit, RMSEA = 0.028, GFI = 0.988, CFI = 0.992, suggesting that the present model supports the three latent factors of the ARS (Fig. 2).

Table 1 shows the difference in the mean score of psychological resilience and coping style between subgroups of the students by their gender, major and grade levels. The total scores on the Asian Resilience Scale ranged from 19 to 95, with a mean of 70.41 (SD = 9.91). There was no significant difference in the resilience level between male and female students (*p* = 0.384). Similarly, there was no significant difference between medical and non-medical (*p* = 0.682) or junior and senior students (*p* = 0.771).

**Table 1:** Mean scores of psychological resilience and coping styles by the undergraduates’ characteristics

Characteristics	Psychological Resilience			SCSQ-PS		
	Mean (SD)	t	p	Mean (SD)	t	p
**Gender**						
Male	70.10 (10.79)	−0.871	0.384	23.92 (5.08)	−4.428	**< 0.001**
Female	70.56 (9.61)			25.11 (5.31)		
**Major**						
Medical	70.49 (10.21)	0.409	0.682	25.03 (5.36)	2.788	**0.005**
Non-medical	70.30 (9.49)			24.32 (5.13)		
**Grade**						
Junior	70.34 (10.11)	−0.292	0.771	24.53 (5.28)	−1.585	0.113
Senior	70.48 (9.67)			24.93 (5.24)		

*SCSQ-PS* Simplified Coping Style Questionnaire-Positive Styles, *SD* Standard deviation, *t* t value, *p p* value

The total score on the SCSQ-PS ranged from 6 to 36, and the mean score was 24.72 (SD = 5.27). Male undergraduate students scored significantly lower than female undergraduate students on the SCSQ-PS (mean score: 23.92 for males, 25.11 for females, *p* < 0.001). Medical students scored significantly higher (mean = 25.03) than their non-medical peers (mean = 24.32) on the SCSQ-PS (*p* = 0.005, Table 1). There was no significant difference in coping style between junior and senior students (*p* = 0.113).

### Correlations between positive coping styles and other factors

The correlation analysis results between positive coping styles and other factors among the students are presented in Table 2. Positive coping styles had a positive correlation with psychological resilience in the total score (r = 0.524, *p* < 0.001) and in each of the factors of the resilience scale (r = 0.506, 0.468, 0.419 for mood control, self-plasticity and coping flexibility, respectively, p < 0.001). The positive coping style significantly correlated with gender (r = 0.106, p < 0.001) and major (r = − 0.067, *p* = 0.003), with females and medical students scoring higher on positive coping than males and non-medical students. There was no significant correlation between grade level and positive coping styles among students (r = 0.038, *p* = 0.057).

**Table 2:** Correlations between positive coping styles and other factors

	Gender	Major	Grade	Self-plasticity	Mood control	Coping flexibility	Total resilience
Positive coping styles	0.106***	−0.067**	0.038	0.468***	0.506***	0.419***	0.524***
Gender		−0.157***	0.009	0.066**	0.051*	−0.058**	0.022
Major			−0.109***	−0.013	− 0.022	0.012	− 0.010
Grade				0.017	0.001	0.005	0.007
Self-plasticity					0.719***	0.638***	0.855***
Mood control						0.703***	0.930***
Coping flexibility							0.877***

* p < 0.05; ** p < 0.01; *** p < 0.001

### Relationship between psychological resilience, students’ demographic characteristics and positive coping styles

Table 3 presents the multiple regression result for the relationship between positive coping styles and the predictor variables. Overall, the model showed a significant association between the independent variables and positive coping style (F = 141.896, *p* < 0.001). Gender (β = 0.85, p < 0.001), major (β = − 0.50, *p* = 0.023), and three factors of resilience, including self-plasticity (β = 0.35, p < 0.001), mood control (β = 0.34, p < 0.001) and coping flexibility (β = 0.14, *p* = 0.002), were significantly associated with a positive coping style. A higher level of resilience was related to higher positive coping style scores. Medical and female students had higher scores for positive coping styles than non-medical students and male students, respectively. There were no multicollinearity problems (Durbin-Watson value = 1.914; tolerance limit = 0.380–0.975; variance inflation factor (VIF) = 1.026–2.630), suggesting that all the exposure variables in the model were independent predictors of the positive coping style. There was no significant interaction between each component of the resilience scale and students’ gender, major and grade (*p* > 0.05) thus the main effect models are presented in Table 3. The regression result for each of the steps (*n* = 5) was showed in the Additional file (see Table, Additional file 1). The variables entered into the model in each step of the multiple regression were as follows: step one: mood control; step 2: mood control and self-plasticity; step 3: mood control, self-plasticity and gender; step 4: mood control, self-plasticity, gender and coping flexibility; step 5: mood control, self-plasticity, gender, coping flexibility and major.

**Table 3:** Regression analysis results for the association between psychological resilience, demographic characteristics and positive coping styles

Variable	Unstandardized coefficient (β)	SE	Standardized coefficient (B)	p	Collinearity statistics	
					Tolerance	VIF
**Psychological resilience**						
Mood control	0.34	0.04	0.30	**< 0.001**	0.380	2.630
Self-plasticity	0.35	0.06	0.19	**< 0.001**	0.445	2.246
Coping flexibility	0.14	0.04	0.09	**0.002**	0.459	2.179
**Gender**						
Female vs. male	0.85	0.23	0.08	< 0.001	0.951	1.051
**Major**						
Non-medical vs. medical	−0.50	0.22	−0.05	**0.023**	0.975	1.026

*SE* Standard error, *p p* value, *VIF* Variance inflation factor; vs.: versus. The reference group for gender and major was male and medical students, respectively

## Discussion

The present study examined the relationship between psychological resilience, students’ characteristics and positive coping styles in a large provincial sample of undergraduates in China. We found that undergraduates with a higher total resilience score and better mood control, self-plasticity and coping flexibility experienced more positive coping styles. Medical and female students had higher scores for positive coping styles than non-medical students and male students. The findings suggest that undergraduates with higher levels of psychological resilience, medical majors and females may exhibit more positive behaviours to cope with stressful and adverse psychological problems than their peers with lower psychological resilience, non-medical majors and males.

The study observed a significant correlation between positive coping style and three components of psychological resilience. The regression analysis showed that the resilience components of mood control, self-plasticity and coping flexibility were all related to positive coping styles; mood control appeared to be the strongest predictor of positive coping style. The results of this study are consistent with previous research showing that students who had better mood control and coping flexibility showed significantly more effective coping strategies when faced with negative psychological and mental health problems [51]. The importance of mood control lies in the control and management of emotional changes. An individual’s change in emotions may be followed by a change in attitudes about life and surroundings [53]*.* Thus, the key to mood control lies in controlling individuals’ attitudes towards their life and surroundings. Prior research has reported that individuals who adopted a positive coping style had better mental health than individuals who adopted a negative coping style [54]. Students with high psychological resilience could more clearly understand the significance of a positive coping style and effectively overcome the influence of negative emotions on themselves in adversity [36]. Additionally, our observation of the positive effect of self-plasticity and coping flexibility on the positive coping style seems consistent with previous studies showing that students with higher resilience were more likely to use problem-solving strategies and avoid an escape from stressful events [44, 55]. The present study’s findings suggest that promoting psychological resilience may help to increase university students’ ability to adopt positive coping styles when experiencing stressful situations.

This study found a significant difference in positive coping styles between genders: males’ positive coping style scores were lower than those of females. The finding is in line with some previous studies showing that female students used different adaptive coping strategies [40, 56]. One explanation for this difference may be that females may use more social support and emotion-focused coping than males [40]. A study by Liu and colleagues indicated that female college students were more likely than male students to use help-seeking behaviours [56]. In addition, with the continuous development and flourishing of the economy and education in China, university students experience increased pressures from society, schools and families. The economic development may increase public awareness of students’ psychological problems among females; thus, paying more attention to the education of positive coping styles among females than males. Male students may be more affected by pressures because they lack a social support and have insufficient experience to adopt positive coping strategies.

The present study contributes to the existing research by revealing a difference in the coping style between medical and non-medical undergraduate students. We found that the positive coping style scores of medical students were higher than those of non-medical students. A possible explanation for the difference may be related to differences between them in the knowledge and skills of coping strategies for mental health problems. Although there are free mental health services, compulsory mental health education and mental health elective courses [56], compared with non-medical students, medical students have more mental health content and practical skills in their curriculum. In addition, there is a component on psychosomatic disease in the main course of medical students, which may make them more aware of the importance of mental health and provide more knowledge and skills of coping strategies than is available to non-medical students. Systematic and diversified psychological course training enables medical students to actively understand the methods for identifying and handling psychological problems and choose more positive coping styles to achieve the goal of alleviating psychological stress. Previous research has documented that compared with the curriculum of medical students, the content of psychological education courses for non-medical students tends to be monotonous and insufficient and cannot satisfy the needs of students [57]. This may also be the reason for the low tendency of non-medical students to choose positive coping styles. Further research is needed to confirm the findings in this study to strengthen the evidence of differences in coping styles among university students in different disciplines. The results suggest that in future mental health courses, different mental health counselling and education content should be formulated according to the characteristics of the students by gender and major.

Currently, the psychological health of Chinese undergraduates has attracted the country’s attention. China is in a special period of social and economic development, with reforms in the economy and education and expanded open policies in foreign collaborations. Chinese undergraduates are burdened with high pressure because of the rapid development of the economy and education and the high expectations of society and their parents. Undergraduates are prone to additional stressors, such as living far from parents, crowded living spaces and strict school regulations [15]. Since the beginning of the twenty-first century, the government has gradually placed greater emphasis on college students’ mental health education [56]. The Chinese Ministry of Education released the ‘Guidelines for Mental Health Education for Students in Colleges and Universities’ [58], which emphasized the necessity and importance of establishing sound college mental health education. A survey of 38 colleges in Beijing found that all colleges provided free psychological counselling services and elective courses, and approximately 78.95% of colleges had compulsory mental health courses [56]. However, mental health counselling services have not aroused widespread public attention due to the influence of traditional concepts (e.g., mental illness stigma) [59, 60]. The public’s mental health awareness is low, and students with mental health problems often deny these problems or do not seek psychological services and counselling. The findings of this study highlight the need for broader investments to promote psychological resilience to foster effective coping styles among undergraduates. As both psychological resilience and coping styles are modifiable and can be changed through scientific interventions [61, 62], intervention programmes to combat negative psychological problems and emotional and mental disorders and to improve the mental health of undergraduate students should focus on enhancing their resilience and nurturing positive coping styles. In the current challenging time of the global pandemic of COVID-19, the World Health Organization has advised positive coping strategies to address various stressful and mental health concerns among adults, youth and children [63]. Mental health counsellors and health professionals for university and college students should provide preventive programmes that integrate the promotion of resilience and positive coping skills in the treatment and prevention of psychological problems.

The primary aim of this study was to assess the relationship between resilience and positive coping styles with regard to their potential protective effects on mental health and the implications for associated interventions among university students. It is worth mentioning that students’ perceived or actual existing stress may also affect their use of coping strategies and resilience, and impact psychological well-being [15, 55, 64, 65]. Interrelations may exist between psychological stress, coping style, resilience and psychological well-being [55, 64, 65]. Future research is needed to better elucidate the role of coping styles and resilience in the relationship between stress and psychological well-being among university students.

The strengths of this study include the use of a large sample representing undergraduates in the province of Shandong, the inclusion of both medical and non-medical undergraduates, and the use of multiple regression analysis to adjust the effect of confounding variables.

The study also has certain limitations. Due to the cross-sectional design, the study cannot establish a causal relationship between psychological resilience and positive coping style. A prospective longitudinal design and experimental studies are needed to demonstrate causality. In addition, the data came from universities in Shandong Province; thus, the present results are likely difficult to generalize to undergraduates in other regions in China. Although the reliability and validity of the measurement scales in the study are high, we cannot exclude the possibility of recall or report bias due to subjective self-reports. Future research is needed to study the coping styles and psychological resilience of undergraduates in other regions and to collect prospective data among undergraduates to support the findings in this study and confirm the directionality between psychological resilience and coping styles.

## Conclusions

Understanding the relationship between undergraduate students’ coping style and psychological resilience is crucial for the mental health and long-term development of undergraduates. The current study observed a significant association between psychological resilience, gender, and major and positive coping styles among undergraduates. The results suggest that strengthening psychological resilience among undergraduates may help increase their tendency to adopt positive coping styles and eventually achieve the goal of promoting mental health and psychological well-being. Further research is needed to illustrate the causal relationship and mechanism between psychological resilience and coping style.

## Supplementary information

**Additional file 1.** Table Stepwise regression results for the final multiple linear regression model.

## Data Availability

The data analysed in this study are not publicly available due to privacy policies but are available from the corresponding author on reasonable request. For further information about data access, please contact the corresponding author, Ying Wang, by email.

## Abbreviations

ARS: Asian Resilience Scale
SCSQ: Simplified Coping Style Questionnaire
SCSQ-PS: Simplified Coping Style Questionnaire-Positive Styles
CFA: Confirmatory factor analysis
RMSEA: Root-mean-square error of approximation
GFI: Goodness-of-fit index
CFI: Comparative fit index
VIF: Variance inflation factor
