# Supplementary material for: Psychological resilience and positive coping styles among Chinese undergraduate students: a cross-sectional study
Source: BMC Psychol. 2020 Aug 6;8:79. doi: 10.1186/s40359-020-00444-y (PMC7406959; doi:10.1186/s40359-020-00444-y)
Supplement: Supplementary file 1 — Additional file 1. Table Stepwise regression results for the final multiple linear regression model. [file 40359_2020_444_MOESM1_ESM.doc]

**Table Stepwise regression results for the final multiple linear regression model**

| **Model steps** | **Variable entered** | **Unstandardized Coefficients** | | **Standardized Coefficients** | **t value** | **p value** | **Collinearity Statistics** | |
| --- | --- | --- | --- | --- | --- | --- | --- | --- |
|  |  | **β** | **SE** | **Beta** |  |  | **Tolerance** | **VIF** |
| Step 1 | Mood control | .573 | .023 | .506 | 24.475 | < .001 | 1.000 | 1.000 |
| Step 2 | Mood control | .397 | .033 | .351 | 11.966 | < .001 | .483 | 2.070 |
|  | Self-plasticity | .403 | .055 | .216 | 7.379 | < .001 | .483 | 2.070 |
| Step 3 | Mood control | .397 | .033 | .350 | 11.990 | < .001 | .483 | 2.070 |
|  | Self-plasticity | .395 | .055 | .212 | 7.245 | < .001 | .482 | 2.074 |
|  | Gender | .827 | .228 | .074 | 3.632 | < .001 | .996 | 1.004 |
| Step 4 | Mood control | .344 | .037 | .303 | 9.247 | < .001 | .380 | 2.628 |
|  | Self-plasticity | .347 | .057 | .186 | 6.127 | < .001 | .445 | 2.245 |
|  | Gender | .932 | .230 | .083 | 4.057 | < .001 | .974 | 1.027 |
|  | Coping flexibility | .134 | .044 | .092 | 3.079 | .002 | .459 | 2.179 |
| Step 5 | Mood control | .342 | .037 | .302 | 9.200 | < .001 | .380 | 2.630 |
|  | Self-plasticity | .348 | .057 | .186 | 6.149 | < .001 | .445 | 2.246 |
|  | Gender | .851 | .232 | .076 | 3.666 | < .001 | .951 | 1.051 |
|  | Coping flexibility | .136 | .043 | .093 | 3.120 | .002 | .459 | 2.179 |
|  | Major | -.495 | .217 | -.047 | -2.280 | .023 | .975 | 1.026 |

*Dependent variable: positive coping style; VIF: variance inflation factor; SE: standard error.*
